# Reduced Pseudocholinesterase Levels Are Associated With Alterations in Lipid Biomarkers and Liver Enzymes Among Ghanaian Male Cocoa Farmers Using Organophosphate Pesticides

**DOI:** 10.1155/tswj/5926091

**Published:** 2026-04-19

**Authors:** Justice Afrifa, Godfred Appiah Kubi, Richard Armah, Eric Ofori Gyamerah, Sandra Ofori, Ibrahim Neveh-Fio, Gabriel Pezahso Kotam, Enoch Essiam, Helena Acquah, Ibrahim Anyass Goumboundi, Samuel Essien-Baidoo, Richard Kobina Dadzie Ephraim

**Affiliations:** ^1^ Department of Medical Laboratory Science, School of Allied Health Science, College of Health and Allied Sciences, University of Cape Coast, Cape Coast, Ghana, ucc.edu.gh; ^2^ Kidney Research Initiative, Cape Coast, Ghana; ^3^ Department of Biology Education, Faculty of Science Education, University of Education, Winneba, Ghana, ue.edu.pk; ^4^ National Vaccine Institute, Accra, Ghana

**Keywords:** agrochemicals, cholinesterase, kidney, lipids, liver, organophosphates

## Abstract

**Background:**

Agrochemical usage is common with cocoa farmers in Ghana; however, the inappropriate use or lack of protective equipment leaves farmers exposed to these chemicals. Pesticides are generally taken up by farmers through inhalation, ingestion, or dermally and distributed through the circulatory system to affect various organs. Organophosphate inhibits cholinesterase (ChE), causing a buildup of acetylcholine and overstimulation of cholinergic synapses. Plasma pseudocholinesterase (pChE), synthesized in the liver, serves as a biomarker for organophosphate exposure.

**Aim:**

We assessed the levels of serum cholinesterase and liver and kidney function biomarkers among cocoa farmers exposed to organophosphates.

**Methods:**

A total of 220 male farmers with a consistent track record of using agropesticides for at least 1 year or more were selected. A structured questionnaire was used to gather sociodemographic information. Following an overnight fast, 5 mL of blood was collected from each participant aseptically for assessment of biochemical markers of the liver, kidney, lipids, and cholinesterase. Reduced plasma pseudocholinesterase was defined as levels < 5000 U/L and estimated glomerular filtration rate (eGFR) was calculated using the chronic kidney disease epidemiology collaboration equation.

**Results:**

The prevalence of reduced pseudocholinesterase (pChE) among the study participants was 23%. The mean level of aspartate aminotransferase (AST) (38.11 ± 14.15 vs. 35.72 ± 16.61, *p* = 0.036) and alanine aminotransferase (ALT) (29.04 ± 18.85 vs. 23.69 ± 11.09, *p* = 0.017) were significantly elevated among subjects with low serum cholinesterase levels. Triglycerides (TG) (1.68 ± 0.86 vs. 1.30 ± 0.66, *p* = 0.004) and very low‐density lipoprotein (VLDL) (0.64 ± 0.35 vs. 0.77 ± 0.40, *p* = 0.004) were significantly elevated among the participants with low pChE levels. However, mean high‐density lipoprotein (HDL) (1.22 ± 0.44 vs. 1.37 ± 0.35, *p* = 0.017) was significantly reduced among participants with low pChE levels. Coffee consumption OR = 2.24 [1.04–4.83, *p* = 0.039], duration of agro pesticide usage greater than 10 years OR = 4.70 [1.72–13.5, *p* = 0.003], and poor knowledge of the harmful effect of pesticides OR = 4.96 [1.97–14.1, *p* = 0.001] were all significantly associated with low pChE levels among the study participants. pChE levels showed a significantly negative correlation with ALT (*R* = −0.2, *p* = 0.0027), TG (*R* = −0.34, *p* ≤ 0.001), and VLDL (*R* = −0.31, *p* ≤ 0.001). HDL showed a significant positive correlation (*R* = 0.14, *p* = 0.044).

**Conclusion:**

There is a high prevalence of reduced pChE among cocoa farmers in Ghana and this was associated with alteration in liver and lipid biomarkers. Additionally, coffee intake, longer work duration, and poor knowledge of agropesticide side effects were associated with low pChE levels among the study participants. These findings highlight the importance of targeted occupational health interventions, including improved training on pesticide safety and consistent and proper use of personal protective equipment (PPE).

## 1. Introduction

Pesticides are widely used chemicals with unique properties designed to control pests and prevent plant disease. Insecticides, herbicides, fungicides, and rodenticides are only a few of the diverse types of pesticides that can be identified. Despite their many advantages, the use of pesticides poses a serious risk to the general public and the environment [1]. Africa is one of the continents with the lowest rates of agrochemical usage worldwide [2]. This could be attributed to several factors such as the subsistence nature of farming and their inability to purchase agrochemicals due to limited financial resources [3]. Only 2%–4% of pesticides are used in Africa, which accounts for less than 5% of the global pesticides business [4], however, farmers in Africa frequently employ unsafe chemical practices despite the decreased use of agrochemicals [5]. Various factors that influence the unsafe use of agrochemicals among these farmers include small farm sizes that force farmers to use agrochemicals directly and the normalization of agrochemicals by smallholder farmers due to their lack of knowledge about safety precautions [5]. The majority of African farmers utilize basic farm equipment like buckets, brushes, brooms, and knapsack sprayers to apply agrochemicals since they lack the financial means and economic incentives to purchase expensive farm gear [5].

One of the most important economic sectors in Ghana is agriculture, which contributes 54% of the country′s total GDP, 40% of export revenue, and more than 90% of the nation′s food requirements [6]. In 2015 Ghana′s Environmental Protection Agency (EPA) indicated that some 540 different kinds of chemicals had been registered for usage in public health and agriculture [7]. Studies conducted by Nwona‐Kwakye and Hogarh (2005) revealed that about 70%–85% of farmers in Ghana use agrochemicals, with vegetable and cash crop farmers using the most [8]. Thus, the rising use of agrochemicals by farmers with limited knowledge of safety procedures and a lack of personal protective equipment is a public health concern [9]. It is therefore important that the use of agrochemicals is regulated, but in Ghana, this is largely impeded by a lack of personnel and logistical support [10].

Several classes of pesticides are deemed dangerous for humans. Among the many different varieties that are readily available, organophosphates, organochlorines, and carbamates are the most frequently utilized [11]. These products′ principal active components are primarily carbamates, organophosphates, chlorinated hydrocarbons, and derivatives of carbamides. Although they have been employed as chemical warfare agents, organophosphate compounds are frequently utilized in agriculture as an insecticide [11]. The health effects of pesticide exposure depend on the chemical makeup of the pesticide, the mode of exposure (skin absorption, ingestion, or inhalation), and the frequency, duration, and severity of exposure [12]. Acute intoxications can result from short‐term high‐dose pesticide exposure, but long‐term low‐dose exposure may mostly cause chronic consequences [13]. Exposure to organophosphates causes organophosphate poisoning. Symptoms include miosis, bradycardia, emesis, sweating, salivation, lacrimation, blurred vision, urine, and feces [14]. Organophosphate and carbamate primarily work by inhibiting the cholinesterase enzyme (ChE), which causes symptoms of excessive cholinergic stimulation [15]. Cholinesterase exists in two different forms: Acetylcholinesterase and pseudocholinesterase. Acetylcholinesterase is found in the RBCs and nerve tissue, whereas pseudocholinesterase is found in the liver and plasma [16]. Organophosphate inhibits pseudocholinesterase, causing a buildup of acetylcholine and overstimulation of cholinergic synapses [14]. Acetylcholine, which is primarily found in the neurological system, skeletal muscle motor end plates, and human erythrocytes, is hydrolyzed by cholinesterase [17].

Pesticides are generally taken up by farmers through inhalation, ingestion, or dermally, and distributed through the circulatory system to affect various organs. The Liver and kidneys are mostly credited as detoxification centers for organisms and are thus vulnerable to xenobiotic derangements [18]. It is also known that chronic exposure to small amounts of organophosphate leads to deleterious effects on carbohydrate metabolism [19] and as such several organs such as the pancreas, liver, muscles, and brain that are involved in the metabolism of carbohydrates, fat, and protein can be affected by pesticides through alterations in glycolysis, glycogenesis, and gluconeogenesis [19]. Despite the widespread use of organophosphate pesticides among cocoa farmers in Ghana, there is limited data linking biochemical evidence of exposure to alterations in liver, renal, and lipid metabolism. We therefore sought to assess the extent of organophosphate exposure by assessing pseudocholinesterase levels and its effect on liver and kidney function, and lipid profile among Ghanaian male cocoa farmers.

## 2. Materials and Methods

### 2.1. Study Area

The research was conducted in the Konongo municipality, specifically focusing on the localities of Adiembra, Chichiwere, Jiasi, and Adumasa. Konongo serves as the capital of the Asante Akim North Municipal and is situated in the Ashanti region, approximately 53 km away from Kumasi. It shares its eastern border with the Kwahu South District in the Eastern Region, whereas the northern boundary adjoins the Sekyere East District, the southern boundary neighbors the Asante Akim South District, and the western boundary connects with the Ejisu‐Juaben Municipal. The total land area of Konongo covers 1160 km^2^, accounting for approximately 4.5% of the Ashanti region′s overall size. Its geographical coordinates are Latitude: 6° 37 ^′^ 0.01“ N and Longitude: 1° 13 ^′^ 0.01” W. Agriculture plays a pivotal role in the municipality′s economy, employing approximately 70% of its population. Among the agricultural activities, about 29.6% of the local farmers cultivate maize, 24% are engaged in cocoa farming, whereas the remaining focus is on cultivating crops such as cassava, plantain, yam, and other staple food crops.

### 2.2. Study Design and Population

In this comparative cross‐sectional study, a purposive sampling method was employed to recruit a total of 220 male farmers with a consistent track record of using agropesticides. Cocoa farmers were selected because cocoa farming involves frequent and intensive use of organophosphate pesticides due to persistent attack by pests [20]. Further, cocoa is a perennial tree crop, which means the farmers are likely to spray more than once in a year and this is often done under limited protective conditions, increasing the likelihood of chronic exposure. A structured questionnaire was pretested using the test‐retest method to ascertain its reliability and was reviewed by experts to ensure content validity. The questionnaires were used to gather socio‐demographic information including age and level of education, their patterns of agropesticide utilization, safety precautions taken, and the symptoms they had experienced while using agropesticide products.

### 2.3. Sample Size Determination

The sample size was estimated using Cochran’s formula

n=Z2p1−pe²

where *n* is the required sample size, *Z* is the *z*‐score for the confidence level (95%), which is 1.96, *p* is the estimated prevalence of organophosphate spraying and *e* is the margin of error.

Since prior prevalence data among Ghanaian cocoa farmers spraying organophosphate pesticides is lacking, a prevalence rate of 50% (0.5) was assumed to maximize the sample with a margin of error of 0.05.

n=1.962∗0.510.5−0.05²

This yielded a minimum sample size of 384 participants. However, due to field‐based constraints, including availability of eligible participants within the study period and voluntary participation, a total of 220 cocoa farmers were recruited. This sample size was considered adequate for detecting statistically significant associations based on similar studies [21].

### 2.4. Inclusion Criteria

The study included only male cocoa farmers aged 15–74 years who had been using agropesticides consistently for at least 1 year prior to the study. The participants were also required to be healthy and without any previously diagnosed chronic diseases. Female cocoa farmers were excluded due to their low representation in our study population after pilot studies.

### 2.5. Sample Collection and Storage

The questionnaire was administered through face‐to‐face interviews by trained research assistants. Following an overnight fast, 5 mL of blood was collected aseptically by trained medical laboratory scientists from each participant into a serum separator tube. The sample was allowed to clot after which the serum was separated by centrifugation (at 3000 rpm for 5 min) and stored at a temperature of −4°C overnight (less than 24 h) [22] for further assessment of biochemical markers of the liver, kidney, lipids, and cholinesterase. The samples were analyzed at the biochemistry department and the research laboratory at the Komfo Anokye Teaching Hospital, Ghana.

### 2.6. Determination of Pseudocholinesterase Levels

Five (5) parts (1000 *μ*L) of the buffer solution were mixed with 1 part (200 *μ*L) of the substrate reagent. The frozen sample was thawed in a water bath at a temperature of 37°C to obtain the liquid serum. A total of 1000 *μ*L of the working reagent was pipetted into the sample cuvette followed by 20 *μ*L of the sample (serum). Analysis was then carried out using the Selectra Pro S fully automated clinical chemistry analyzer (Elitech, United States). The cutoff for reduced cholinesterase levels was set at < 5000 U/L [23]. Those with reduced cholinesterase (pChE < 5000 U/L) were classified as cases, whereas those with pChE above 5000 U/L were classified as controls. The assay performance was verified according to manufacturer specifications, including acceptable limits for analytical precision and accuracy. All reagents used were supplied by Elitech Clinical Systems (United States) and used according to the manufacturer′s instruction.

### 2.7. Analysis of Biochemical Markers

The assessment of liver functioning biomarkers namely aspartate aminotransferase (AST), alanine aminotransferase (ALT), GGT, ALP, total protein, serum albumin, total bilirubin, and globulin was performed with Vitros reagent packs (slides) on a Vitros 5600 Integrated Analyzer. Renal biomarkers including Urea, creatinine were analyzed with the Vitros 5600 Integrated Analyzer. Serum lipid biomarkers such as Total cholesterol, triglyceride, VLDL, LDL, and HDL were measured with the Vitros 5600 Integrated Analyzer (QuidelOrtho Corporation, San Diego, United States).

### 2.8. eGFR Estimation

The chronic kidney disease epidemiology collaboration (CKD‐EPI) equation was used to estimate the eGFR [24], where serum creatinine, age, and sex were incorporated into the formula to estimate kidney function expressed as mL/min/1.73m^2^. This was calculated automatically by the computer based on measured serum creatinine values, age, and sex.

### 2.9. Data Processing and Analysis

The data obtained was entered into a Microsoft Excel sheet and statistically analyzed using R software. Continuous variables were summarized using means and standard deviations or medians and interquartile ranges (IQR) as appropriate. Categorical variables were presented as frequencies and percentages. Independent sample *t*‐tests were used to compare mean biochemical parameters between groups. Multivariable logistic regression analysis was performed to identify factors independently associated with reduced plasma pseudocholinesterase levels. A *p* value < 0.05 was deemed statistically significant.

## 3. Results

**Figure 1:**
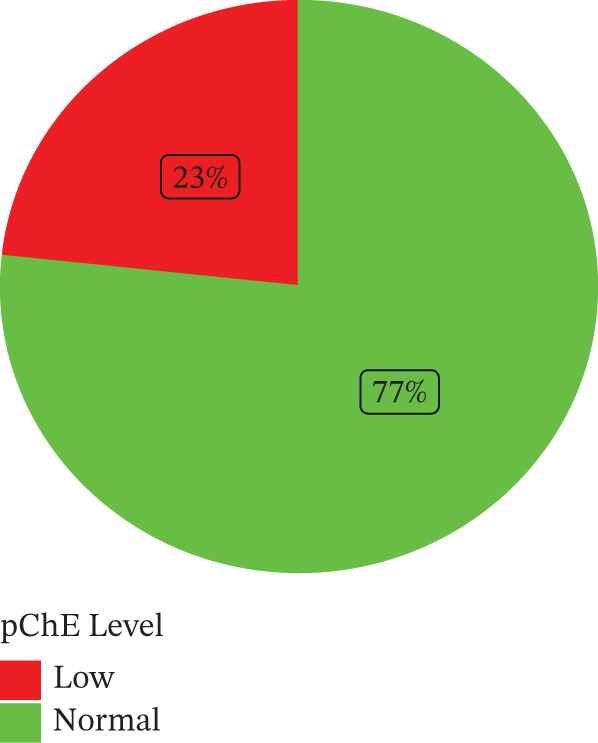
Prevalence of reduced pChE levels among the study participants.

The prevalence of reduced pCHE among the study participants was 23% (Figure 1). The median age of the cohort was 39 years, with IQR spanning from 33 to 46 years. There was no significant difference in the median age (*p* = 0.5) when those with reduced pChE were compared with the normal group. Among the study participants, the most represented age group was between 35–54 years (63%). Participants were moderately educated as two‐thirds of the participants (66%) had completed Junior high school education. Alcohol consumption (69%) was relatively high among the participants compared with cigarette smoking (8.6%) and coffee consumption (26%). It is important to note that coffee consumption was associated with pChE levels (*p* = 0.044); however, alcohol consumption was not significantly associated with pChE levels (*p* = 0.3). More than half of the participants had used pesticides within 1–5 years. About 75% of the participants reported the use of PPEs, whereas 78% of them reported consistent use of PPEs. Most of the participants (66%) had poor knowledge of the adverse effects associated with pesticide use. The results indicated knowledge about pesticide use is significantly associated with pChE levels (*p* = 0.031) among the study participants (Table 1).

**Table 1:** Sociodemographic characteristics of study participants stratified by pChE (U/g Hb) < 5000 CHT.

Characteristic	Overall, *N* = 220^1^	Low, *N* = 51^1^< 5000	Normal, *N* = 169^1^≥ 5000	*p* value^2^
**Age (years)**	39 (33, 46)	38 (32, 48)	40 (34, 46)	0.5
**Age group**				0.11
15–34	64 (29%)	20 (39%)	44 (26%)	
35–54	139 (63%)	26 (51%)	113 (67%)	
55–74	17 (7.7%)	5 (9.8%)	12 (7.1%)	
**Education level**				0.8
Uneducated	16 (7.3%)	4 (7.8%)	12 (7.1%)	
Junior high school	145 (66%)	32 (63%)	113 (67%)	
Senior high school	58 (26%)	15 (29%)	43 (25%)	
Tertiary	1 (0.5%)	0 (0%)	1 (0.6%)	
**Smoking status**				0.2
No	201 (91%)	44 (86%)	157 (93%)	
Yes	19 (8.6%)	7 (14%)	12 (7.1%)	
**Coffee consumption**				0.044
No	162 (74%)	32 (63%)	130 (77%)	
Yes	58 (26%)	19 (37%)	39 (23%)	
**Alcohol consumption**				0.3
No	97 (44%)	26 (51%)	71 (42%)	
Yes	123 (56%)	25 (49%)	98 (58%)	
**PPE usage**				0.5
No	56 (25%)	15 (29%)	41 (24%)	
Yes	164 (75%)	36 (71%)	128 (76%)	
**Frequency of PPE usage**				0.2
Always	172 (78%)	37 (73%)	135 (80%)	
Sometimes	47 (21%)	13 (25%)	34 (20%)	
Never	1 (0.5%)	1 (2.0%)	0 (0%)	
**Duration of agro pesticide usage (years)**				0.2
1–5 years	125 (57%)	26 (51%)	99 (59%)	
6–10 years	50 (23%)	10 (20%)	40 (24%)	
> 10 years	45 (20%)	15 (29%)	30 (18%)	
**Knowledge on adverse effect**				0.031
Good	75 (34%)	11 (22%)	64 (38%)	
Poor	145 (66%)	40 (78%)	105 (62%)	

^1^Median (IQR); *n* (%).

^2^Wilcoxon rank sum test; fisher′s exact test; Pearson′s chi‐squared test.

Table 2 Shows biochemical markers with significant variations between reduced pChE and normal pChE levels. Mean levels of AST (38.11 ± 14.15 vs. 35.72 ± 16.61, *p* = 0.036) and ALT (29.04 ± 18.85 vs. 23.69 ± 11.09, *p* = 0.017) were significantly elevated among subjects with low pChE levels compared with those with normal pChE levels. For lipids, the results indicated that triglycerides (1.68 ± 0.86 vs. 1.30 ± 0.66, *p* = 0.004) and VLDL (0.64 ± 0.35 vs. 0.77 ± 0.40, *p* = 0.004) were significantly elevated among the participants with low pChE levels compared with the subjects with normal pChE levels. However, mean HDL (1.22 ± 0.44 vs. 1.37 ± 0.35, *p* = 0.017) was significantly reduced among participants with low pChE levels compared with those with normal pChE levels.

**Table 2:** Comparison of means of biochemical markers stratified according to pChE levels.

Biochemical markers	Overall, *N* = 220^1^	Low, *N* = 51^1^	Normal, *N* = 169^1^	*p* value^2^
pChE (U/g Hb)	7564.71 (2975.36)	3267.41 (1162.08)	8861.53 (1959.74)	< 0.001
AST (U/L)	36.27 (16.07)	38.11 (14.15)	35.72 (16.61)	0.036
ALT (U/L)	24.93 (13.44)	29.04 (18.85)	23.69 (11.09)	0.017
ALP (U/L)	89.18 (25.19)	90.71 (24.69)	88.72 (25.40)	0.5
GGT (U/L)	38.18 (20.57)	40.86 (19.46)	37.37 (20.88)	0.10
TP (g/dL)	80.97 (7.38)	81.62 (6.99)	80.78 (7.50)	0.7
ALB (g/dL)	40.98 (3.15)	41.02 (3.91)	40.97 (2.90)	0.7
GLO (g/dL)	40.03 (6.34)	40.78 (5.78)	39.80 (6.50)	0.5
TB (mg/dL)	17.16 (7.65)	16.85 (6.01)	17.26 (8.09)	0.7
DB (mg/dL)	5.68 (4.13)	5.93 (2.42)	5.61 (4.53)	0.2
IDB (mg/dL)	11.47 (7.67)	10.92 (6.40)	11.64 (8.03)	0.8
Total cholesterol (mg/dL)	4.09 (0.85)	3.99 (0.82)	4.11 (0.86)	0.5
Triglycerides (mg/dL)	1.39 (0.73)	1.68 (0.86)	1.30 (0.66)	0.004
HDL (mg/dL)	1.34 (0.38)	1.22 (0.44)	1.37 (0.35)	0.017
LDL (mg/dL)	2.12 (0.89)	1.94 (0.74)	2.17 (0.93)	0.2
VLDL (mg/dL)	0.64 (0.35)	0.77 (0.40)	0.60 (0.32)	0.004
Urea (mg/dL)	4.58 (1.20)	4.70 (1.21)	4.55 (1.20)	0.5
Creatinine (mg/dL)	79.04 (10.72)	77.71 (10.97)	79.44 (10.64)	0.6
BUN (mg/dL)	28.40 (21.42)	27.97 (7.40)	28.53 (24.11)	0.4
eGFR	119.75 (14.00)	120.80 (15.37)	119.43 (13.59)	0.7

^1^Mean (SD); *n* (%).

^2^Wilcoxon rank sum test; Pearson′s chi‐squared test.

Abbreviations: ALB, albumin; BUN, blood urea nitrogen; DB, direct bilirubin; GLO, globulin; IDB, indirect bilirubin; TB, total bilirubin; TP, total protein.

A multivariate logistic regression of factors associated with low pChE levels among the study participants shows that coffee consumption (OR = 2.24 [95% CI = 1.04–4.83], *p* = 0.039), duration of agropesticide usage greater than 10 years (OR = 4.70 [95% CI = 1.72–13.5], *p* = 0.003), and poor knowledge of the harmful effect of pesticides (*OR* = 4.96 [95% CI = 1.97–14.1], *p* = 0.001) were all significantly associated with low pChE levels among the study participants (Table 3).

**Table 3:** Univariate and multivariable logistic regression analysis of factors associated with low pChE (U/g Hb) < 5000 cholinesterase levels.

	Univariate			Multivariable		
Characteristic	OR^1^	95% CI^1^	*p* value	OR^1^	95% CI^1^	*p* value
Age group						
15–34	—	—		—	—	
35–54	0.49	0.25, 0.98	0.043	0.47	0.22, 1.01	0.054
55–74	0.90	0.26, 2.78	0.85	0.71	0.17, 2.63	0.6
Education level						
Uneducated	—	—		—	—	
Junior high school	0.85	0.27, 3.20	0.79	1.05	0.30, 4.34	> 0.9
Senior high school	1.05	0.31, 4.19	0.94	1.29	0.33, 5.89	0.7
Smoking status						
No	—	—		—	—	
Yes	2.27	0.79, 6.12	0.11	2.46	0.79, 7.40	0.11
Coffee consumption						
No	—	—		—	—	
Yes	2.03	1.03, 3.97	0.039	2.24	1.04, 4.83	0.039
Alcohol consumption						
No	—	—		—	—	
Yes	0.70	0.37, 1.32	0.27	0.60	0.29, 1.20	0.15
PPE usage						
No	—	—		—	—	
Yes	0.77	0.39, 1.59	0.47	1.07	0.49, 2.43	0.9
Duration of agro pesticide usage (years)						
1–5 years	—	—		—	—	
6–10 years	0.94	0.40, 2.08	0.89	1.42	0.54, 3.57	0.5
> 10 years	1.88	0.87, 3.99	0.10	4.70	1.72, 13.5	0.003
Knowledge on adverse effect						
Good	—	—		—	—	
Poor	2.24	1.10, 4.87	0.032	4.96	1.97, 14.1	0.001

^1^OR, odds ratio, CI, confidence interval.

pChE levels showed a significantly negative correlation with ALT (*R* = −0.2, *p* = 0.0027), TG (*R* = −0.34, *p* ≤ 0.001), and VLDL (*R* = −0.31, *p* ≤ 0.001). AST showed no significant correlation (*R* = −0.13, *p* = 0.052) with pChE, whereas HDL showed a significant positive correlation (*R* = 0.14, *p* = 0.044), Figure 2.

Figure 2Correlation of biochemical markers with pChE levels among the study participants.

**Figure:**
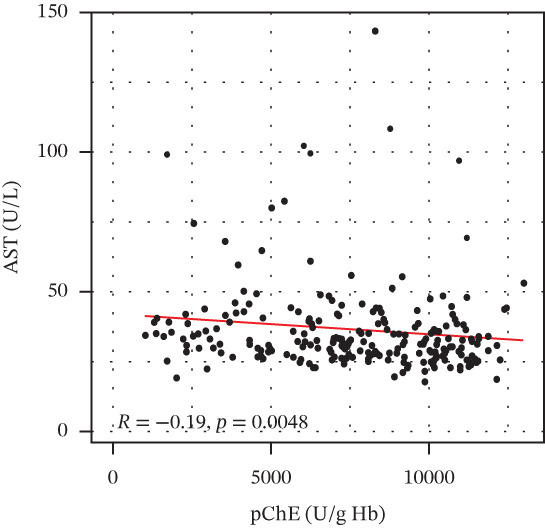
(a)

**Figure:**
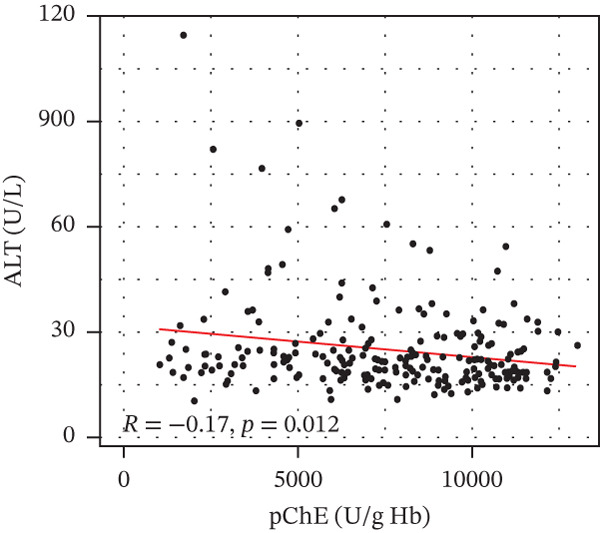
(b)

**Figure:**
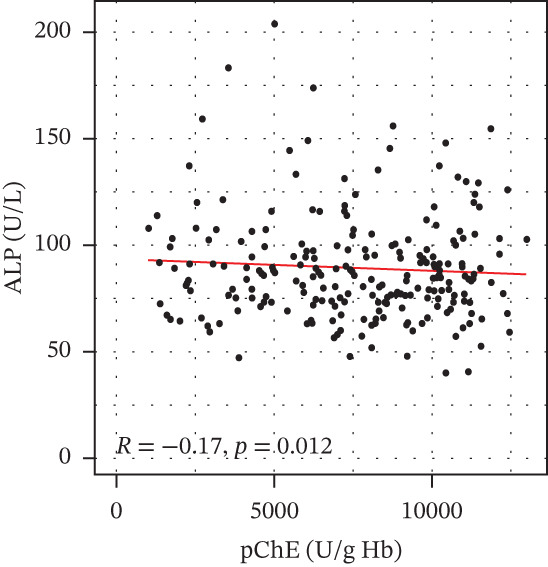
(c)

**Figure:**
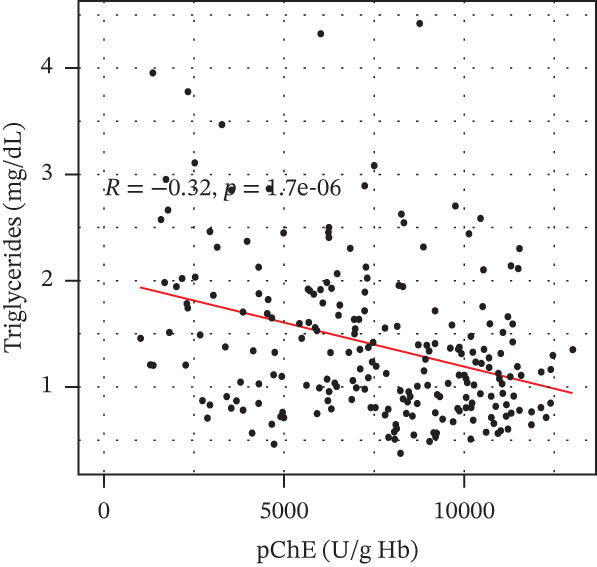
(d)

**Figure:**
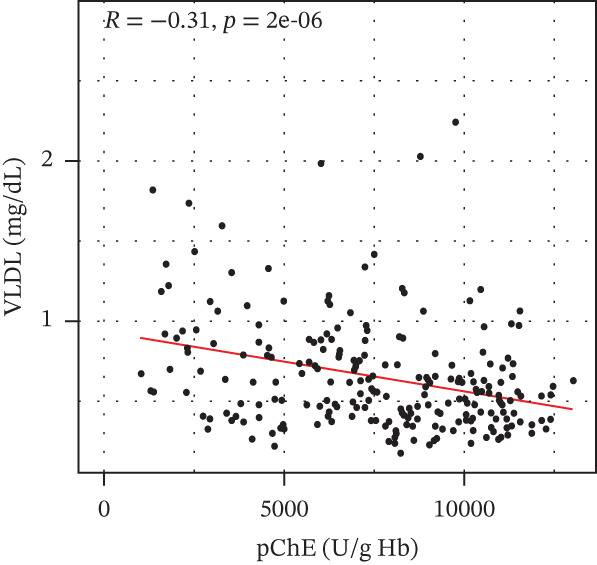
(e)

**Figure:**
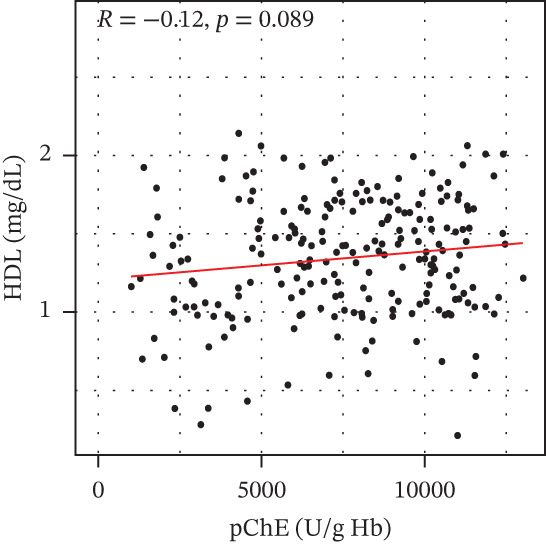
(f)

## 4. Discussion

Prolonged exposure to organophosphates causes excess stimulation to the muscles, exocrine glands, and postsynaptic neurons, which might have negative effects [25]. Consequently, pChE activity levels have been employed as a reliable biomarker for organophosphate pesticide exposure [26–28]. Measuring plasma pseudocholinesterase levels can help determine the severity and likelihood of acute exposures [25] and severe poisoning is indicated by reduced plasma pseudocholinesterase levels. In the current study, we sought to assess the extents of organophosphate exposure by assessing pseudocholinesterase levels and its effect on liver and kidney function, and lipid profile among Ghanaian male cocoa farmers.

Our results indicated a high prevalence of reduced pChE levels among the study participants. Further, liver enzymes, AST and ALT levels were elevated among subjects with reduced pChE. Among the lipid profile markers, we observed high levels of TG and VLDL with low levels of HDL in subjects with reduced pChE. Finally, coffee consumption, duration of agro pesticide usage greater than 10 years, and poor knowledge of the harmful effect of pesticides were all significantly associated with low pChE levels among the study participants.

Among the participants of our study who are all organophosphate users, 23% recorded reduced levels of pChE below the normal threshold (Figure 1). Compared with our study, Neupane and Co reported the prevalence of low pChE levels to be 8.51% among organophosphate users, albeit a smaller sample size of 25 farmers, which could account for the lower prevalence they observed [29]. These findings are in line with other studies that have shown that high exposure to organophosphates leads to reduced pChE activity [30–32].

We observed a median age of 33 years, with a greater percentage of our participants (66%) having some form of formal education. On the contrary, a cross‐sectional investigation conducted on 240 Ghanaian farmers had an average age of 52 years, with a higher percentage (3.3%) obtaining a university degree compared with what was observed (1%) in our current study [33]. This observation could contribute to the generally poor knowledge of farmers (66%) on the adverse effects of exposure to organophosphates, observed in this study.

Serum cholinesterase is synthesized in the liver and lower levels tend to be associated with liver dysfunction [34]. Therefore, pChE serves as an important marker for the prognosis of liver dysfunction. Consequently, we observed a significant increase in both AST (*p* = 0.036) and ALT (*p* = 0.017) among the participants with reduced pChE levels compared with their counterparts with normal pChE levels. Again, with a cutoff of 40 U/L and 41 U/L for AST and ALT, respectively, the prevalence of elevated AST and ALT among the study participants was 24.9% and 30.9%, respectively, which indicates an alteration in liver function biomarkers among the study participants. Various studies have shown a negative association between pChE and liver enzymes [35, 36], and specifically among organophosphate pesticide exposed horticulturists, elevation of liver enzymes including AST and ALT were observed [37].

Physiologically, the role of pChE in mammalian organisms has been associated with the metabolism of lipids and lipoproteins [38]. The relationship between lipid metabolism and serum cholinesterase level among organophosphate‐exposed subjects seems inconclusive due to the variation in available reports. Although earlier studies have reported an association of a reduced pChE level with reduced total cholesterol and triglyceride levels [23], findings from this study, similar to the reports of Pothu et al. [39] indicate high levels of triglycerides and VLDL and low HDL levels among participants with reduced pChE compared with those with normal pChE levels. It is also important to note that triglycerides and VLDL correlated negatively with pChE, whereas HDL correlated positively with pChE. These results support findings of earlier studies that suggest that chronic exposure to organophosphate pollutants could lead to subjects developing metabolic syndrome [40]. Further, xenobiotic substances are usually implicated in the activation of the sympathetic nervous system resulting in epinephrine and norepinephrine release from the adrenal medulla [41]. These activate tissue hormone‐sensitive triglyceride lipase, which hydrolyses stored fat from the adipose tissue leading to elevated free fatty acid in the blood stream [42].

We observed no significant decrease or increase in any of the renal markers measured, similar to the study by Pothu and Co [39]. This implies that variations in pChE levels might not always result in appreciable changes in renal function indicators. This may be because of minimal exposure or because most (75%) of the farmers in this study reported that they were frequent users of PPE. Compensatory mechanisms within the body as well as specificities of cholinesterase inhibitors to different organs could also contribute to the lack of correlation. In exposed populations, therefore, there is a need to explore alternative markers for a more comprehensive assessment of health.

Lastly, we observed that coffee consumption, usage of pesticides for 10 years or more, and poor knowledge of the adverse effects of organopesticide usage were indicative of exposure and reduced pChE levels among our study participants. Although there is evidence to show that coffee consumption can weakly inhibit pChE activity [43], other studies have reported no inhibitory relationship between coffee consumption and pChE activity [44]. However, in an in vitro study, pChE activity was inhibited using fractionated coffee extracts digested in the gastrointestinal tract [45]. Xenobiotics like organophosphates are usually insidious in nature and hence may take a longer time for the necessary accumulation to make an impact on organ function; thus, people who have been exposed for a long time are more likely to experience severe adverse effects, as shown in this study. These findings support the outcome of previous studies that have established an association between long‐term organophosphate exposure and pChE inhibition [46, 47] Again, the lack of knowledge on the adverse effect of organophosphate exposure could influence the right use of PPEs and the right application of protocols, which may affect the levels of exposure.

## 5. Conclusion

This study demonstrates a high prevalence of reduced pChE among cocoa farmers in Ghana and this was associated with alteration in liver and lipid biomarkers. Again, coffee intake, longer work duration, and poor knowledge of agropesticide side effects were associated with low pChE levels among the study participants. This study contributed valuable data on organophosphate poisoning among at‐risk groups. Even though the majority of the participants reported consistent use of PPEs, the level of organophosphate poisoning among the participants is still considerably high, which may raise the question of education on the proper and safe use of these PPE. Thus, consistent education and training of such participants on safe use of these PPEs could be crucial in reducing the adverse effects of these organophosphates. It is also important that the local government agencies in charge of agriculture facilitate the subsidization of costs and acquisition of PPEs to ensure accessibility. Also, it is recommended that larger cohort studies should be conducted to elucidate the actual state of organophosphate poisoning among users to prevent any adverse effects.

**Nomenclature**
ALP: alkaline phosphatase
ALT: alanine transaminase
AST: aspartate transaminase
ChE: Cholinesterase
CKD‐EPI: chronic kidney disease epidemiologic collaboration
eGFR: estimated glomerular filtration rate
GGT: gamma glutamyl transferase
HDL: high‐density lipoprotein
pChE: pseudocholinesterase
TG: triglyceride
VLDL: very low‐density lipoprotein

## Funding

No funding was received for this manuscript.

## Disclosure

All authors have read and approved the final version of the manuscript. JUSTICE AFRIFA had full access to all of the data in this study and takes complete responsibility for the integrity of the data and the accuracy of the data analysis.

## Ethics Statement

Ethical clearance was sought from the University of Cape Coast Institutional Review Board (UCCIRB/CHAS/2023/198). Both written and verbal consent was sought from individual participants before the commencement of the study.

## Conflicts of Interest

The authors declare no conflicts of interest.

## Data Availability

Data will be made available upon request from the corresponding author.
